# Dietary Polysaccharides and Gut Microbiota Ecosystem

**DOI:** 10.3390/nu14204285

**Published:** 2022-10-14

**Authors:** Ana I. Álvarez-Mercado, Julio Plaza-Diaz

**Affiliations:** 1 Instituto de Investigación Biosanitaria ibs.GRANADA, Complejo Hospitalario Universitario de Granada, 18014 Granada, Spain; 2 Department of Biochemistry and Molecular Biology II, School of Pharmacy, University of Granada, 18071 Granada, Spain; 3 Institute of Nutrition and Food Technology, Biomedical Research Center, University of Granada, 18016 Armilla, Spain; 4 Children’s Hospital of Eastern Ontario Research Institute, Ottawa, ON K1H 8L1, Canada

The intestinal microbiota is a community of microorganisms that subsists within the gastrointestinal ecosystem. In human health, the role of the gastrointestinal microbiota is to maintain a dynamic balance with the host. This balance plays both local and remote roles in critical physiological processes, particularly inflammation, and the immune response [1].

Natural polysaccharides are polymeric carbohydrate macromolecules and sources of fermentable dietary fiber. Polysaccharides are the most abundant dietary components in the gut microbiota and are deeply involved in host health [2]. Emerging evidence shows the involvement of polysaccharides in numerous functions in gut microbiota-host symbiosis, such as microbial interactions with endogenous host glycans, and the key role of microbial polysaccharides [3]. Additionally, bacterial polysaccharides act as immunomodulators, and host-derived polysaccharides protect host cells from pathogenic microbial neighbors and affect overall gut health through interactions with gut microbes. The growth of certain beneficial intestinal bacteria can be promoted by polysaccharides (among other things) during intestinal fermentation, changing the microbiota profile of the gut and altering both local and remote host physiology, which can reduce disease development [3,4].

This special issue includes eight papers, seven of them are original publications [5,6,7,8,9,10,11], and one review [12]. Overall, these works highlight mechanisms involving changes in microbiota affected by polysaccharides as well as the evaluation of dietary polysaccharides–health links.

For instance, using a rat model of liver transplantation in steatotic and non-steatotic livers from donors after brain death, Micó-Carnero et al. (2021) [9] found that lipid treatment is an effective nutritional support, better than glucose, to protect against hepatic damage in steatotic liver transplantation from donors after brain death. The observed benefits are due to reductions in intestinal damage and, consequently, preservation of the gut microbiota [9].

In animals, but in this instance in a mouse model of metabolic syndrome, Ejima et al. (2021) [8] showed that seaweed dietary fiber sodium alginate suppresses the migration of colonic inflammatory monocytes and diet-induced metabolic syndrome through the gut microbiota [8].

In humans, studies included in this special issue cover different stages in life. In a study conducted on Mexican children and adults, Martínez-Medina et al. (2021) [6] examined whether microbial enterotypes modify the association between dietary fiber intake and metabolic traits. Their results suggest that individuals harboring a *Prevotella*-dominant enterotype may have a more pronounced benefit on insulin resistance markers upon consumption of hemicellulose-rich foods [6].

Fiber intake also induced changes in a population of Spanish children who consumed infant cereals differing in whole grain and sugar content as first weaning foods. Results from this study indicated a supporting effect of infant cereals with 50% whole grains and a reduced sugar content over infant cereals manufactured with refined hydrolyzed flours on the infant microbiota [11].

The fact that diet is a determinant of bodyweight and gut microbiota composition was supported by the results reported by Rodríguez-Lara et al. (2022) [5]. In this study performed in a subpopulation of young Mexican adults, the authors found changes in several gut microbes after fiber consumption. These changes indicated that a higher fiber intake had a positive impact on the body and mediates positive changes in gut microbiota [5].

Another intervention study was conducted on healthy adults and mice fed with fermented *Brassica rapa* L. extract. The oral administration of this vegetable could be responsible for modulating the gut microbiota to increase fiber-degrading bacteria and butyrate-producing bacteria [10].

Under the hypothesis that fine differences in carbohydrate linkage structure would govern microbial community structure and function independently of variation in glycosyl residue composition, Romero-Marcia et al. (2021) [7] fermented commercially available soluble resistant glucans, composed of glucose linked in different structural arrangements, in vitro with human fecal inocula. Their findings revealed that variation in linkage structure independently of sugar composition governs compositional and functional responses of microbiota [7].

Finally, the review included in this collection presents a summary of recent knowledge regarding how dietary polysaccharides affect gut microbiota composition and host health. The immunomodulatory properties of certain polysaccharides are crucial to the regulation of immune responses during the progression of certain diseases [12]. It is believed that polysaccharides may also have health benefits by modulating the gut microbiota, besides stimulating the growth of certain intestinal bacteria. There has been an exponential increase in studies pertaining to the triad gut microbiota–polysaccharides–health in recent years [12].

The present special issue could be considered a comprehensive summary of the ongoing research into polysaccharides/fibers as modulators of gut microbiota. It is noteworthy that these data highlight the crucial role played by polysaccharides in host–gut microbiota composition and function. Thus, intestinal fiber fermentation is a highly effective tool for modulating microbiota, its functionality, and inflammation.
